# Functional Watermelon Jelly Fortified with Potato By-Product-Derived Single-Cell Protein: Antioxidant and Enzyme Inhibitory Potential with Possible Health-Promoting Properties

**DOI:** 10.3390/molecules31183288

**Published:** 2026-09-16

**Authors:** Milena Rašeta, Mladen Simonović, Fariddudin Mohammadian, Marko Jovanović, Vladimir Nešić, Maja Karaman, Aleksandra Radulović, Nabil Adrar, Gizem Kezer, Tuba Esatbeyoglu

**Affiliations:** 1 Department of Chemistry, Biochemistry and Environmental Protection, Faculty of Sciences, University of Novi Sad, Trg Dositeja Obradovića 3, 21000 Novi Sad, Serbia; 2 Institute of General and Physical Chemistry, Studentski trg 12/V, 11158 Belgrade, Serbia; mladensimonovic@gmail.com (M.S.); markspace711@gmail.com (M.J.); radulovica32@gmail.com (A.R.); 3 Department of Molecular Food Chemistry and Food Development, Institute of Food and One Health, Leibniz University Hannover, 30167 Hannover, Germany; mohammadian@foh.uni-hannover.de (F.M.); adrar@foh.uni-hannover.de (N.A.); kezer@foh.uni-hannover.de (G.K.); esatbeyoglu@foh.uni-hannover.de (T.E.); 4 Faculty of Medicine, University of Belgrade, Dr Subotića 8, 11000 Belgrade, Serbia; vlanesic@gmail.com; 5 Clinic of Otorhinolaryngology and Maxillofacial Surgery, University Clinical Centre of Serbia, 11000 Belgrade, Serbia; 6 ProFungi Laboratory, Department of Biology and Ecology, Faculty of Sciences, University of Novi Sad, Trg Dositeja Obradovića 2, 21000 Novi Sad, Serbia; maja.karaman@dbe.uns.ac.rs

**Keywords:** *Citrullus lanatus*, watermelon jelly, single-cell protein, pectin, antioxidant activity, α-amylase inhibition, α-glucosidase inhibition, functional food, nutraceuticals, bioactive compounds

## Abstract

Watermelon (*Citrullus lanatus*) is a widely consumed fruit rich in carotenoids, phenolic compounds, vitamins, and other bioactive constituents associated with antioxidant and health-promoting properties. The present study aimed to develop novel low-sugar watermelon jelly formulations enriched with pectin and single-cell protein (SCP), and to evaluate their bioactive and functional properties, including the antioxidant and enzyme inhibitory activities of their 70% ethanol (70% EtOH) extracts. Six jelly formulations (W1–W6) differing in pectin and SCP supplementation were prepared using concentrated watermelon pulp processed under low-temperature vacuum conditions to preserve thermolabile bioactive compounds. The total phenolic content (TPC) and total flavonoid content (TFC) were determined in the 70% EtOH extracts using spectrophotometric methods. TPC remained relatively stable among the investigated formulations, with the highest value recorded for sample W6, which contained 0.58 g pectin and 0.215 g SCP (1.37 mg gallic acid equivalents (GAE)/mL extract). The highest TFC was observed for sample W5, corresponding to the formulation supplemented with 0.5 g pectin and 0.300 g SCP (5.87 mg quercetin equivalents (QE)/mL extract). Antioxidant activity, evaluated using ABTS, DPPH, and FRAP assays, demonstrated considerable antioxidant potential in all 70% EtOH extracts. The strongest antioxidant activity was observed in pectin-rich samples, with ABTS and FRAP values reaching 89.06 mg Trolox equivalents (TE)/mL extract and 8.99 mg ascorbic acid equivalents (AAE)/mL extract, respectively. The potential carbohydrate-hydrolyzing enzyme inhibitory activity of the 70% EtOH extracts was assessed through α-amylase and α-glucosidase inhibition assays. The formulation containing both pectin and SCP exhibited the strongest α-amylase inhibitory activity (58.93%), whereas the highest α-glucosidase inhibition (60.54%) was observed in the pectin-containing formulation without SCP supplementation. These findings indicate that both pectin and SCP significantly influenced the nutritional and bioactive properties of the developed watermelon jelly formulations. Overall, the results demonstrate that enrichment with pectin and SCP significantly enhances the nutritional and bioactive properties of watermelon jelly formulations. These findings highlight indicate that pectin and SCP were associated with differences in the bioactive and functional properties of the developed watermelon jelly formulations. Overall, the results demonstrate the potential of pectin- and SCP-enriched watermelon jellies as sustainable functional food products with promising antioxidant and enzyme inhibitory properties.

## 1. Introduction

Watermelon (*Citrullus lanatus* (Thunb.) Matsum. & Nakai, fam. Cucurbitaceae) represents one of the most widely cultivated fruit crops worldwide [1]. Numerous cultivars are grown globally, including many seedless varieties [2]. Cultivated watermelons are typically 40–60 cm in diameter and may exceed 5 kg in weight. Due to its high moisture content (>92%), watermelon is considered an excellent dietary source of water-soluble vitamins, minerals, and health-promoting phytochemicals [3]. Although commonly categorized as a vegetable in culinary practice, watermelon is botanically classified as a fruit and is consumed fresh or incorporated into desserts, salads, syrups, candies, jams, and other processed products [4,5,6,7]. Besides the flesh, watermelon rind and seeds are increasingly recognized as valuable by-products due to their nutritional and functional properties [8]. Watermelon seeds are particularly rich in unsaturated fatty acids and proteins and are commonly consumed after roasting.

Watermelon contains a broad spectrum of biomolecules and phytonutrients, including proteins, sugars, dietary fibers, flavonoids, alkaloids, carotenoids, and antioxidant compounds [9,10,11,12]. Among the major antioxidants identified in watermelon are phenolic compounds, ascorbic acid, and carotenoids, especially lycopene, which is responsible for the characteristic red coloration of the flesh [3,13,14]. Furthermore, watermelon seeds represent an important source of vitamin E and ω-3 fatty acids [15,16]. Watermelon rind is particularly rich in pectic polysaccharides, making it an attractive raw material for the development of functional food products and natural gelling systems [17,18].

In recent years, increasing attention has been directed toward sustainable food processing strategies and the development of nutrient-enriched functional foods using alternative protein sources [19]. Single-cell proteins (SCPs), derived from microbial biomass, have emerged as promising sustainable ingredients due to their high protein content, favorable amino acid composition, rapid production, and relatively low environmental footprint [20]. The incorporation of SCP into food formulations represents an innovative approach for improving nutritional quality while supporting green chemistry and circular bioeconomy principles.

The present study aimed to develop watermelon jelly with a high fruit content and reduced sucrose addition while preserving its bioactive compounds through mild processing conditions, including vacuum-assisted preparation and sonication. Unlike conventional jelly formulations that rely primarily on commercial gelling agents such as pectin or gelatin [21,22,23], this approach explored the formation of gel-like structures under modified processing conditions designed to minimize thermal degradation of sensitive phytochemicals and enhance the release of intracellular bioactive compounds. Although previous studies have utilized watermelon rind (mainly albedo) as a natural source of pectin for jelly production [24,25,26], these formulations often require additional sweeteners to achieve desirable sensory properties [27]. In contrast, the present study focused on the naturally sweet red flesh of watermelon to reduce the need for added sugar while maintaining acceptable product characteristics. Although sucrose was still required to achieve appropriate gel texture and consumer acceptability, its level was lower than that typically reported for conventional jelly formulations. Furthermore, this study represents, to the best of our knowledge, the first investigation of watermelon jelly fortified with SCP, thereby combining fruit processing with the valorization of an agro-industrial by-product in accordance with circular bioeconomy principles. The selected potato by-product-derived SCP, previously developed and characterized by our research group, was incorporated as a sustainable functional ingredient because microbial biomass represents a promising source of proteins and naturally occurring bioactive constituents that may contribute to the functional properties of the developed formulations. The effects of pectin concentration, SCP supplementation, and sonication treatment on the bioactive composition and physicochemical properties of the developed formulations, as well as the antioxidant and *in vitro* α-amylase and α-glucosidase inhibitory activities of their 70% EtOH extracts, were systematically evaluated.

## 2. Results and Discussion

### 2.1. Total Phenolic Content (TPC) and Total Flavonoid Content (TFC)

**Figure 1:**
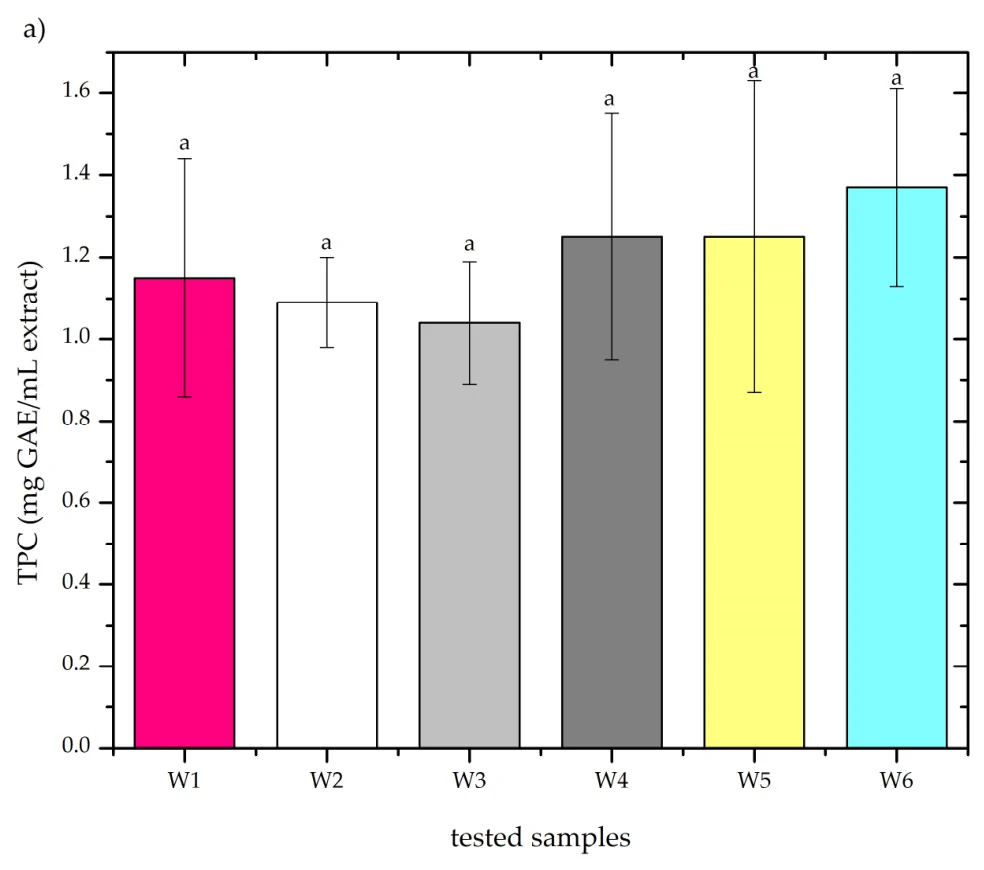

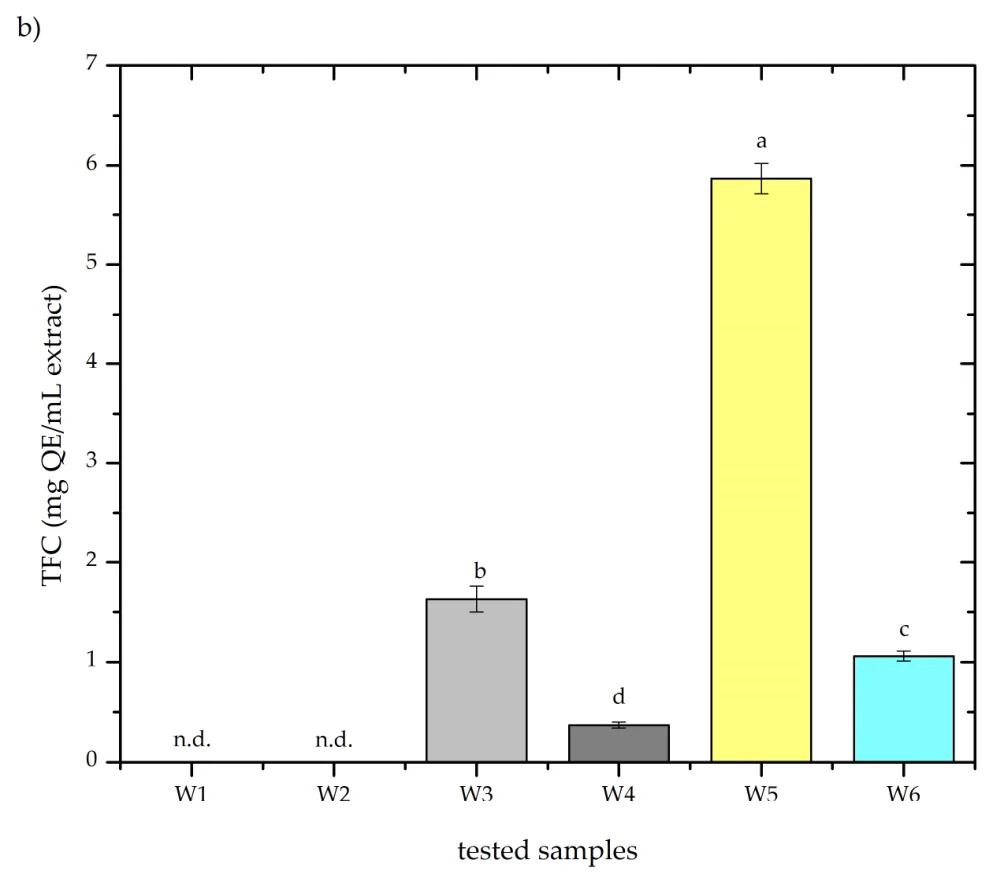
(**a**) Total phenolic content (TPC) and (**b**) total flavonoid content (TFC) of 70% EtOH extracts prepared from watermelon jelly formulations (W1–W6). TPC values are expressed as mg GAE/mL extract, while TFC values are expressed as mg QE/mL extract. Data are presented as mean ± standard deviation (*n* = 3). Different superscript letters indicate statistically significant differences among samples (*p* < 0.05). Abbreviations: W1—control jelly without SCP and pectin; W2—jelly supplemented with 0.5 g pectin; W3—jelly supplemented with 0.58 g pectin; W4—W1 supplemented with 0.215 g SCP; W5—W2 supplemented with 0.300 g SCP; W6—W3 supplemented with 0.215 g SCP; GAE—gallic acid equivalents; QE—quercetin equivalents; n.d.—not detected within the observed quercetin calibration curve range.

The total phenolic content (TPC) and total flavonoid content (TFC) of the 70% EtOH extracts prepared from the formulated watermelon jellies are presented in Figure 1.

The obtained results demonstrated that formulation composition, particularly the addition of pectin and SCP, influenced the phenolic and flavonoid contents of the investigated samples. TPC values ranged from 1.04 to 1.37 mg GAE/mL extract. The W6 sample, containing 0.58 g pectin and 0.215 g SCP, exhibited the highest TPC value (1.37 ± 0.24 mg GAE/mL extract), whereas the W3 sample, formulated with 0.58 g pectin without SCP addition, showed the lowest phenolic content (1.04 ± 0.15 mg GAE/mL extract).

However, one-way ANOVA indicated no significant differences in TPC values among watermelon samples W1–W6 (F = 0.683, *p* = 0.645). The absence of significant differences suggests that neither pectin nor SCP substantially altered the overall TPC of the jelly formulations under the applied processing conditions. Nevertheless, a slight increase in TPC was observed following SCP incorporation, which may be associated with the presence of bioactive metabolites and antioxidative compounds originating from the microbial biomass.

In contrast, statistically significant differences were observed for TFC among the analyzed formulations (F = 908.74, *p* < 0.0001), followed by Tukey’s post hoc test. Unlike TPC, flavonoid content appeared to be more responsive to formulation changes, particularly SCP supplementation, indicating that flavonoid compounds or their interactions within the jelly matrix were more sensitive to the presence of microbial biomass than the total phenolic fraction. Previous studies have shown that SCP preparations may contain peptides, amino acids, vitamins, and other biologically active constituents capable of contributing to the antioxidant potential of functional food formulations [28]. TFC values varied markedly among the investigated watermelon jelly formulations, ranging from 0.37 to 5.87 mg QE/mL extract. The highest TFC was recorded in sample W5, supplemented with 0.5 g pectin and 0.300 g SCP (5.87 ± 0.15 mg QE/mL extract), whereas no detectable flavonoid content was observed in samples W1 and W2, corresponding to the control formulation and the formulation containing only 0.5 g pectin, respectively. Moderate TFC values were detected in samples W3 (1.63 ± 0.13 mg QE/mL extract), W4 (0.37 ± 0.03 mg QE/mL extract), and W6 (1.06 ± 0.05 mg QE/mL extract). One-way ANOVA revealed statistically significant differences among the analyzed formulations (*p* < 0.0001), indicating that SCP incorporation and pectin concentration had a substantial influence on flavonoid accumulation in the developed jellies. The pronounced increase in TFC observed in SCP-enriched samples, particularly in sample W5, may be attributed to the contribution of microbial-derived bioactive metabolites, including phenolic-like compounds and antioxidant constituents naturally present in SCP preparations. Furthermore, interactions between pectin and flavonoid compounds may enhance flavonoid stability and retention within the jelly matrix. Similar findings have been reported in previous studies investigating functional food systems enriched with microbial biomass and hydrocolloid-based stabilizers, where improved flavonoid content and antioxidant properties were observed following SCP supplementation [28].

Collectively, the obtained findings demonstrate that both pectin and SCP can significantly modulate the phenolic and flavonoid contents of watermelon jellies. Although SCP supplementation is expected to increase the total protein content due to its protein-rich composition, and pectin, as a polysaccharide gelling agent, may contribute to an increase in total carbohydrate content, these assumptions were not experimentally verified in the present study because total protein and total carbohydrate contents were not determined. This limitation has been acknowledged in the Section 2.5. and should be considered when interpreting the nutritional implications of the developed formulations. Nevertheless, the results support the potential application of SCP as a sustainable functional ingredient for the development of value-added watermelon jelly products with enhanced antioxidant and enzyme inhibitory properties while preserving naturally occurring bioactive compounds. Due to the lack of directly comparable studies on watermelon products enriched with SCP, the present findings are discussed in the context of the broader literature on watermelon-based products and watermelon-derived bioactive compounds. Previous studies on fruits from the family Cucurbitaceae have mainly focused on the utilization of different fruit parts, such as the albedo, rind, peel, and seeds [24,26,29,30]. Watermelon rind, particularly its white inner part known as the albedo, is relatively rich in nutrients and pectic substances, making it suitable for use in the preparation of products such as marmalades, jams, and jellies [18,31]. However, unlike the red edible flesh of watermelon, the rind/albedo contains a lower amount of sugar, which makes the addition of sweeteners necessary during product formulation. To the best of our knowledge, there are no literature reports on watermelon-based jellies with reduced sugar content, increased protein content, and no extra gelling agents. Krajewska et al. [24] developed vegan jelly candies enriched with powdered watermelon pomace obtained from whole yellow watermelon fruits pressed without prior peeling, meaning that the material contained both edible flesh and parts of the rind. The potential of watermelon-derived materials for the formulation of functional jelly products with improved physicochemical, textural, and antioxidant properties was demonstrated [24]. Marinelli et al. [26] reported that watermelon jelly candies enriched with orange peel flour (containing albedo and flavedo) exhibited increased TPC and enriched antioxidant activity. In contrast, the present study investigated the incorporation of SCP together with pectin as an alternative formulation strategy aimed at developing a functional watermelon jelly with improved bioactive properties. Although SCP is a protein-rich ingredient and pectin is a polysaccharide-based gelling agent, the total protein and carbohydrate contents of the developed formulations were not determined in the present study. Therefore, any potential changes in their nutritional composition remain hypothetical and should be confirmed by future compositional analyses. Nevertheless, the developed formulations retained detectable polyphenols and exhibited considerable antioxidant and enzyme inhibitory activities in their 70% EtOH extracts.

### 2.2. Antioxidant Activity

**Figure 2:**
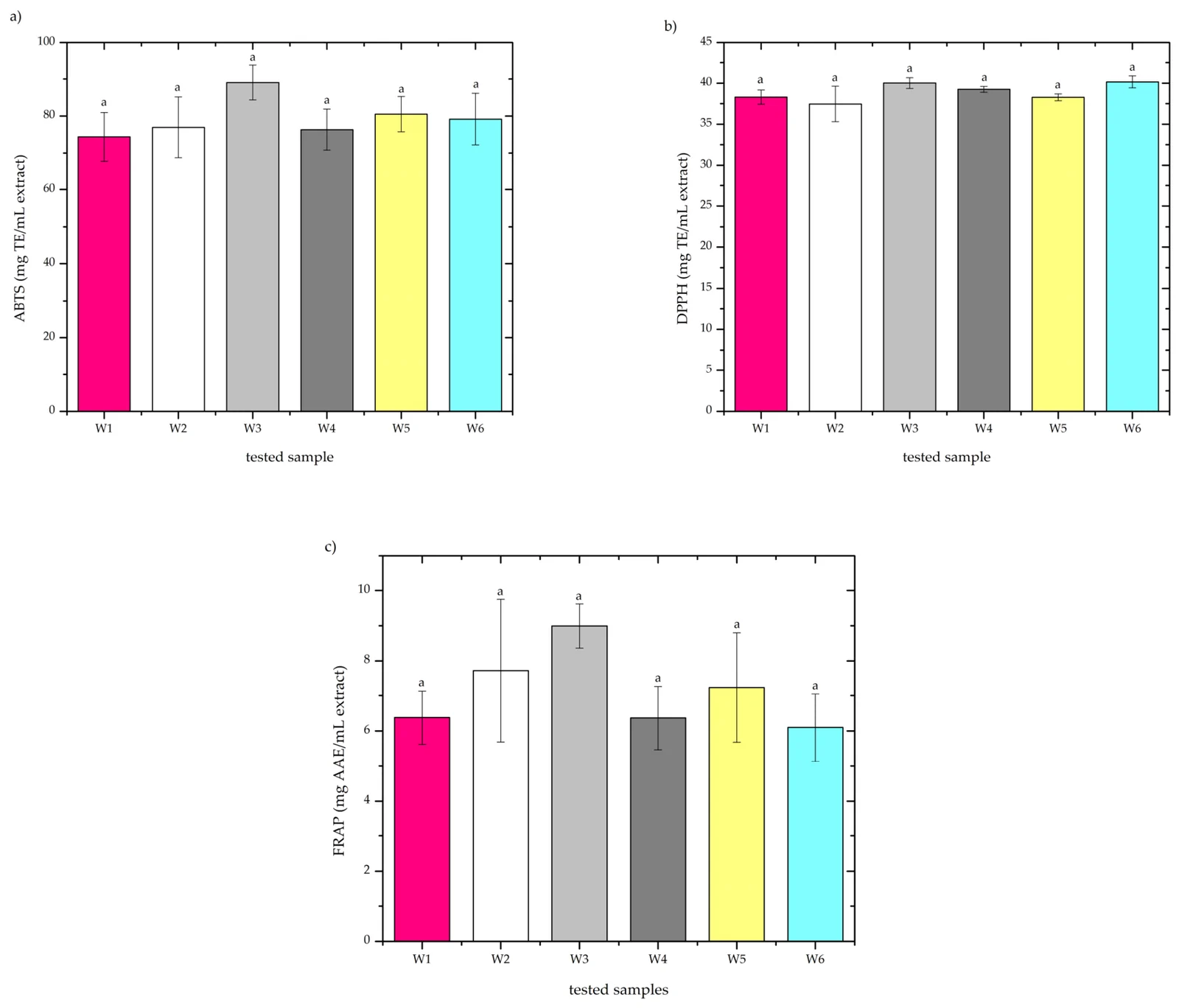
Antioxidant activity of 70% EtOH extracts prepared from watermelon jelly formulations (W1–W6) determined by (**a**) ABTS radical scavenging assay, (**b**) DPPH radical scavenging assay, and (**c**) FRAP assay. ABTS and DPPH values are expressed as mg TE/mL extract, while FRAP values are expressed as mg AAE/mL extract. Data are presented as mean ± standard deviation (*n* = 3). Identical superscript letters indicate no significant differences among samples according to one-way ANOVA followed by Tukey’s post hoc test (*p* > 0.05). Abbreviations: W1—control jelly without SCP and pectin; W2—jelly supplemented with 0.5 g pectin; W3—jelly supplemented with 0.58 g pectin; W4—W1 supplemented with 0.215 g SCP; W5—W2 supplemented with 0.300 g SCP; W6—W3 supplemented with 0.215 g SCP; TE—Trolox equivalents; AAE—ascorbic acid equivalents. All formulations were normalized to a final mass of 8.8 g.

The antioxidant activity of the 70% EtOH extracts from the formulated watermelon jellies was evaluated using ABTS, DPPH, and FRAP assays, and the obtained results are presented in Figure 2.

Overall, the 70% EtOH extracts of all jelly formulations exhibited considerable antioxidant potential, although the extent of activity varied depending on formulation composition, particularly pectin concentration and SCP supplementation.

ABTS radical scavenging activity ranged from 74.35 to 89.06 mg TE/mL extract. The highest ABTS value was observed in sample W3 (89.06 ± 4.69 mg TE/mL extract), corresponding to the formulation containing the highest pectin concentration without SCP addition, whereas sample W1 showed the lowest activity (74.35 ± 6.62 mg TE/mL extract). However, one-way ANOVA indicated no significant differences among the analyzed formulations (F = 2.072, *p* = 0.1398), suggesting that neither pectin nor SCP markedly affected the overall ABTS radical scavenging capacity. The elevated ABTS activity in pectin-rich formulations may be associated with improved stabilization and retention of antioxidant compounds within the jelly matrix. Pectins are known to interact with phenolic compounds through hydrogen bonding and polysaccharide–polyphenol interactions, potentially protecting sensitive antioxidants during processing and storage [32,33]. The comparable ABTS values observed in SCP-enriched formulations further indicate that SCP supplementation did not compromise the antioxidant capacity of the corresponding extracts.

DPPH scavenging activity showed relatively narrow variation among the investigated samples, with values ranging from 37.46 to 40.15 mg TE/mL extract. The highest activity was detected in sample W6 (40.15 ± 0.73 mg TE/mL extract), followed closely by sample W3 (40.01 ± 0.65 mg TE/mL extract), while the lowest value was recorded for sample W2 (37.46 ± 2.17 mg TE/mL extract). Similarly, no statistically significant differences were observed among the formulations (*p* > 0.05), indicating that the modifications in pectin concentration and SCP supplementation had only a minor influence on DPPH radical scavenging activity. The relatively stable DPPH scavenging activity across all samples suggests that the applied processing conditions, including reduced-temperature preparation and sonication, effectively preserved antioxidative constituents within the watermelon matrix. Similarly to the ABTS assay, SCP incorporation did not compromise radical scavenging efficiency, indicating that protein supplementation is compatible with the antioxidant properties of the developed formulations.

Ferric reducing antioxidant power (FRAP) values varied between 6.10 and 8.99 mg AAE/mL per extract. Sample W3 again demonstrated the highest antioxidant potential (8.99 ± 0.63 mg AAE/mL extract), whereas sample W6 exhibited the lowest reducing capacity (6.10 ± 0.96 mg AAE/mL extract). As with the ABTS and DPPH assays, the observed differences were not statistically significant (*p* > 0.05), suggesting that the reducing capacity of the jelly formulations remained largely unaffected by the tested formulation modifications. Nevertheless, the slightly higher FRAP values in formulations containing increased pectin concentrations may indicate improved retention of reducing compounds, including phenolics and ascorbic acid, which are recognized contributors to ferric ion reduction. Watermelon is naturally rich in lycopene and hydrophilic antioxidants, and the mild processing conditions applied in this study likely minimized oxidative degradation of these compounds.

Although no statistically significant differences were observed among the formulations in the antioxidant assays, the consistently high antioxidant activity across all samples demonstrates that the applied processing conditions effectively preserved the bioactive constituents of watermelon. Moreover, neither pectin nor SCP supplementation adversely affected antioxidant capacity, supporting the feasibility of producing protein-enriched watermelon jellies while maintaining their functional properties.

Direct comparison of the obtained antioxidant values with previous studies is limited because antioxidant activity is strongly influenced by the watermelon fraction used, extraction procedure, assay conditions, and the units of expression. Most published studies have focused on products prepared from watermelon rind, pomace, or mixed fruit fractions in combination with commercial gelling agents [6,24,26]. Nevertheless, these studies likewise demonstrated that watermelon-derived materials retain measurable antioxidant activity in gelled food systems, supporting the potential of watermelon as a suitable raw material for the development of functional confectionery products.

The findings of the present study are consistent with previous reports demonstrating that watermelon-derived materials can contribute to the antioxidant properties of gelled confectionery products. Krajewska et al. [24] reported measurable antioxidant activity in vegan jellies enriched with watermelon rind powder, while Tarahi et al. [6] attributed the antioxidant potential of watermelon exocarp to its phenolic compounds, flavonoids, anthocyanins, and vitamin C. Similarly, Marinelli et al. [26] demonstrated that watermelon-derived ingredients can enhance the functional value of confectionery products. Although these studies investigated different watermelon fractions, they collectively support the suitability of watermelon as a source of bioactive compounds for functional jelly formulations. In the present study, 70% EtOH extracts from all formulations exhibited considerable antioxidant capacity, and SCP supplementation did not adversely affect antioxidant properties, whereas pectin-enriched formulations tended to exhibit slightly higher antioxidant values.

### 2.3. α-Amylase and α-Glucosidase Inhibitory Activity

**Figure 3:**
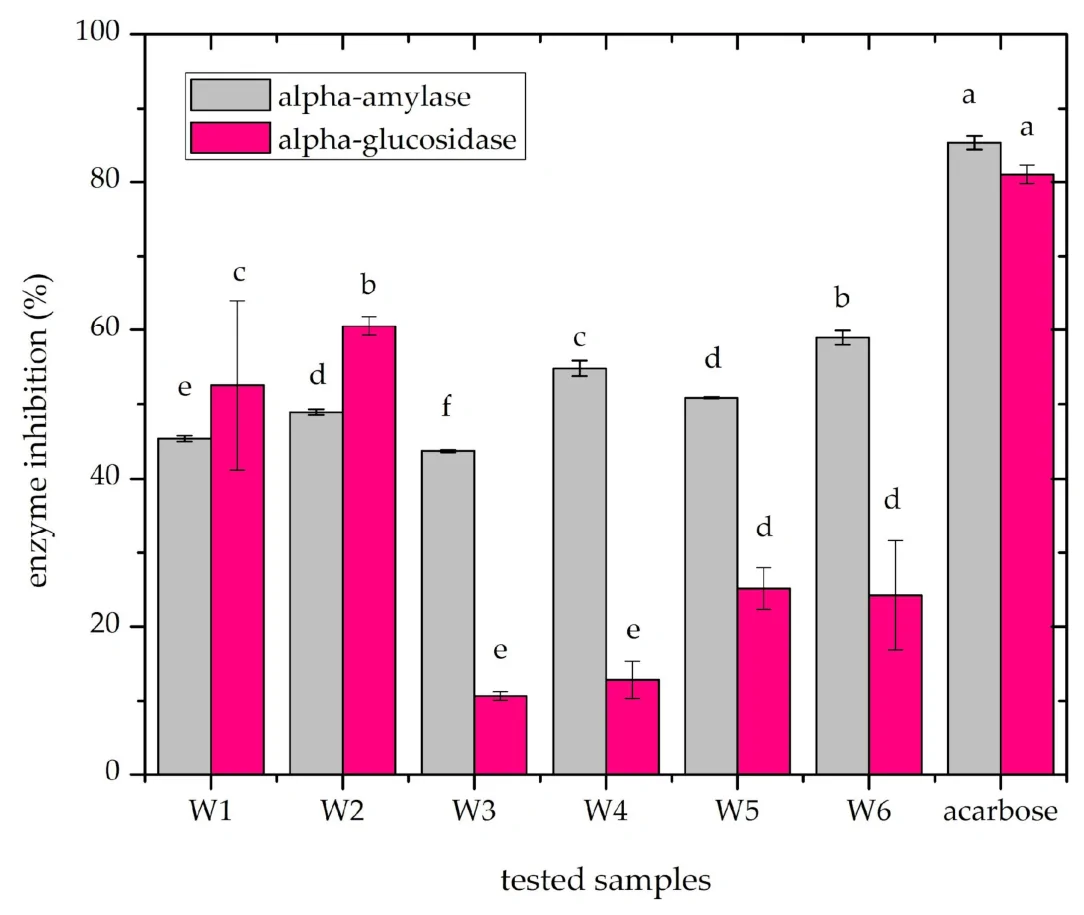
Inhibitory activity of 70% EtOH extracts prepared from formulated watermelon jelly samples against α-amylase and α-glucosidase enzymes. Values are presented as mean ± SD (*n* = 3). Different superscript letters indicate significant differences among samples according to one-way ANOVA followed by Tukey’s post hoc test (*p* < 0.05). Abbreviations: W1—control jelly without SCP and pectin; W2—jelly supplemented with 0.50 g pectin; W3—jelly supplemented with 0.58 g pectin; W4—W1 supplemented with 0.215 g SCP; W5—W2 supplemented with 0.300 g SCP; W6—W3 supplemented with 0.2 g SCP. All formulations were normalized to a final mass of 8.8 g.

The inhibitory potential of 70% EtOH extracts prepared from the formulated watermelon jellies against carbohydrate-hydrolyzing enzymes, α-amylase and α-glucosidase, was evaluated in order to assess their potential antihyperglycemic effects through the inhibition of carbohydrate digestion (Figure 3).

The obtained results demonstrated that formulation composition, particularly the addition of pectin and SCP, markedly influenced the enzyme inhibitory activity of the corresponding extracts.

The α-amylase inhibitory activity of the 70% EtOH extracts ranged from 43.72 to 58.93%. Statistically significant differences among the samples were observed (*p* < 0.0001). The highest inhibition was recorded for sample W6 (58.93 ± 0.96%), containing 0.58 g pectin and 0.215 g SCP per 8.8 g of jelly, while the lowest activity was detected in sample W3 (43.72 ± 0.16%), containing 0.58 g pectin without SCP supplementation. The observed differences among formulations suggest that formulation composition, particularly the incorporation of pectin and SCP, influenced the measured α-amylase inhibitory activity. However, since the individual SCP and pectin preparations were not chemically characterized in the present study, it cannot be excluded that their intrinsic composition, including endogenous enzyme inhibitors, residual carbohydrates, or other bioactive constituents, contributed to the observed effects. Therefore, the underlying mechanisms remain unclear and require further investigation.

The α-glucosidase inhibitory activity of the 70% EtOH extracts showed even greater variation among the analyzed samples, ranging from 10.68 to 60.54%, with statistically significant differences confirmed by one-way ANOVA (*p* < 0.0001). The strongest inhibition was observed in sample W2 (60.54 ± 1.29%), followed by sample W1 (52.58 ± 11.39%), whereas samples W3 and W4 exhibited the lowest inhibitory activities (10.68 ± 0.58% and 12.83 ± 2.50%, respectively). Unlike α-amylase inhibition, the highest α-glucosidase inhibitory activity was not associated with SCP supplementation, indicating that the effects of pectin and SCP differed depending on the target enzyme. These findings suggest that an optimal balance of formulation components, rather than increasing the amount of a single ingredient, is important for maximizing α-glucosidase inhibition. The lower inhibitory activity observed in some SCP-enriched formulations may result from interactions between proteins and bioactive compounds that reduce the accessibility of enzyme inhibitory constituents.

Acarbose, used as the positive control, exhibited the strongest inhibitory activity against both enzymes (85.25 ± 0.91% for α-amylase inhibition and 81.01 ± 1.26% for α-glucosidase inhibition). However, it should be emphasized that acarbose was tested at a substantially lower concentration than the jelly extracts, highlighting its high inhibitory potency as a purified pharmaceutical inhibitor. Nevertheless, several jelly extracts demonstrated considerable inhibitory activity despite being derived from complex food matrices rather than isolated compounds. Overall, the enzyme inhibition results indicate that pectin and SCP affected α-amylase and α-glucosidase differently. While SCP supplementation appeared to enhance α-amylase inhibition, the strongest α-glucosidase inhibition was achieved in formulations containing pectin without excessive SCP supplementation, suggesting that different formulation components contribute to the inhibition of these enzymes through distinct mechanisms.

Direct comparison with previous studies is limited because reports on watermelon jellies enriched with SCP are currently unavailable. Nevertheless, our previous study demonstrated promising α-amylase and α-glucosidase inhibitory activities in mango jelly formulations, suggesting their potential role in glycemic control, highlighting how fruit-based jelly formulations could become functional nutraceutical products for modulating postprandial glucose metabolism [34]. The observed enzyme inhibitory activities may be explained by the combined effects of watermelon-derived bioactive compounds, pectin, and SCP. Pectin, as a soluble dietary fiber, may increase the viscosity of the gastrointestinal contents, delay gastric emptying, and limit enzyme–substrate interactions, thereby moderating postprandial glucose release [35,36,37]. In addition, pectin contributes to the formation of a denser food matrix, which may further reduce the accessibility of carbohydrates to digestive enzymes [36]. SCP contributes to protein enrichment of the formulation; while its proteins and peptides may also contribute to the observed enzyme inhibitory activity [38]. Collectively, these mechanisms are consistent with the experimentally observed enzyme inhibitory activities and support the potential application of SCP-enriched watermelon jellies as functional food products with the potential to modulate carbohydrate digestion and postprandial glucose release.

The combined effects of watermelon-derived bioactive compounds, pectin, and SCP may therefore contribute to the observed enzyme inhibitory activity and the potential of these formulations to modulate carbohydrate digestion [35,36,39].

A limitation of this study is that the obtained results are based solely on *in vitro* α-amylase and α-glucosidase inhibitory assays and therefore indicate only the potential of the developed formulations to modulate carbohydrate digestion. These findings should not be interpreted as evidence of antidiabetic efficacy, which requires confirmation through *in vivo* and clinical studies.

Overall, these findings suggest that watermelon jellies enriched with SCP and pectin represent promising functional food products with potential nutritional and funactional benefits, as reflected by the antioxidant and enzyme inhibitory activities observed in their 70% EtOH extracts. Beyond their favorable technological characteristics, watermelon provides naturally occurring bioactive compounds, including phenolics, flavonoids, carotenoids, and vitamin C, while potato by-product-derived SCP contributes high-quality protein together with microbial bioactive constituents. The combination of these ingredients may provide complementary functional effects through antioxidant activity and modulation of carbohydrate digestion. Although the present findings are limited to *in vitro* antioxidant and enzyme inhibitory assays, they support the potential of these formulations as innovative, sustainable functional foods. Further studies, including detailed phytochemical characterization, bioaccessibility assessments, and *in vivo* investigations, are warranted to confirm their physiological benefits and potential role in promoting metabolic health. Given their demonstrated antioxidant activity, these formulations may warrant future investigation in disorders associated with oxidative stress. Chronic rhinosinusitis, in which oxidative stress is recognized as an important contributor to disease pathophysiology, may represent one potential area for future research [40,41]. However, this possibility remains hypothetical and requires confirmation through dedicated mechanistic, *in vivo*, and clinical studies. In addition, the bioactive constituents naturally present in microbial protein, including peptides and cell-wall polysaccharides [42], may further contribute to the functional potential of the developed formulations, although these effects were not specifically investigated in the present study.

### 2.4. Principal Component Analysis

**Figure 4:**
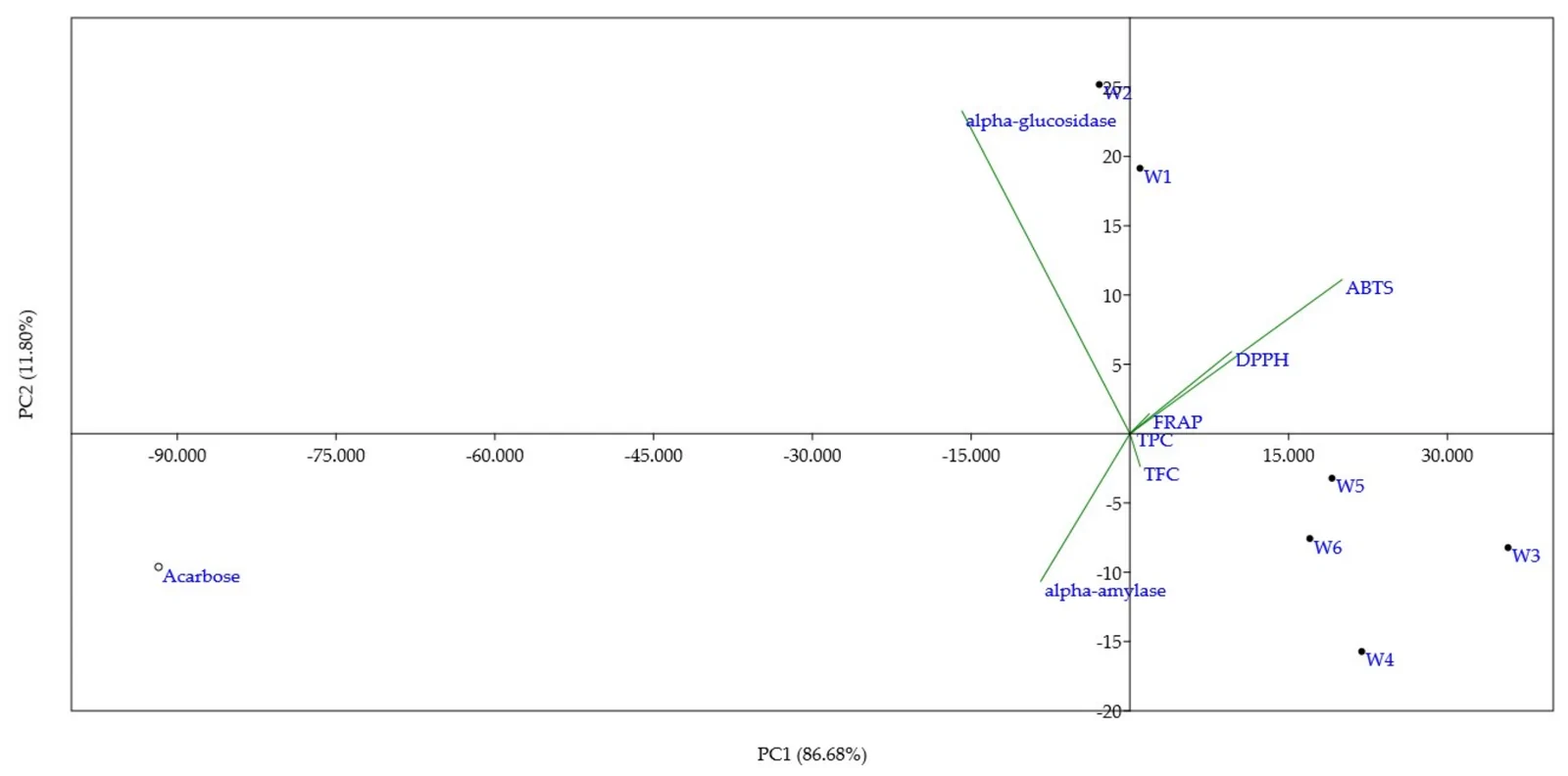

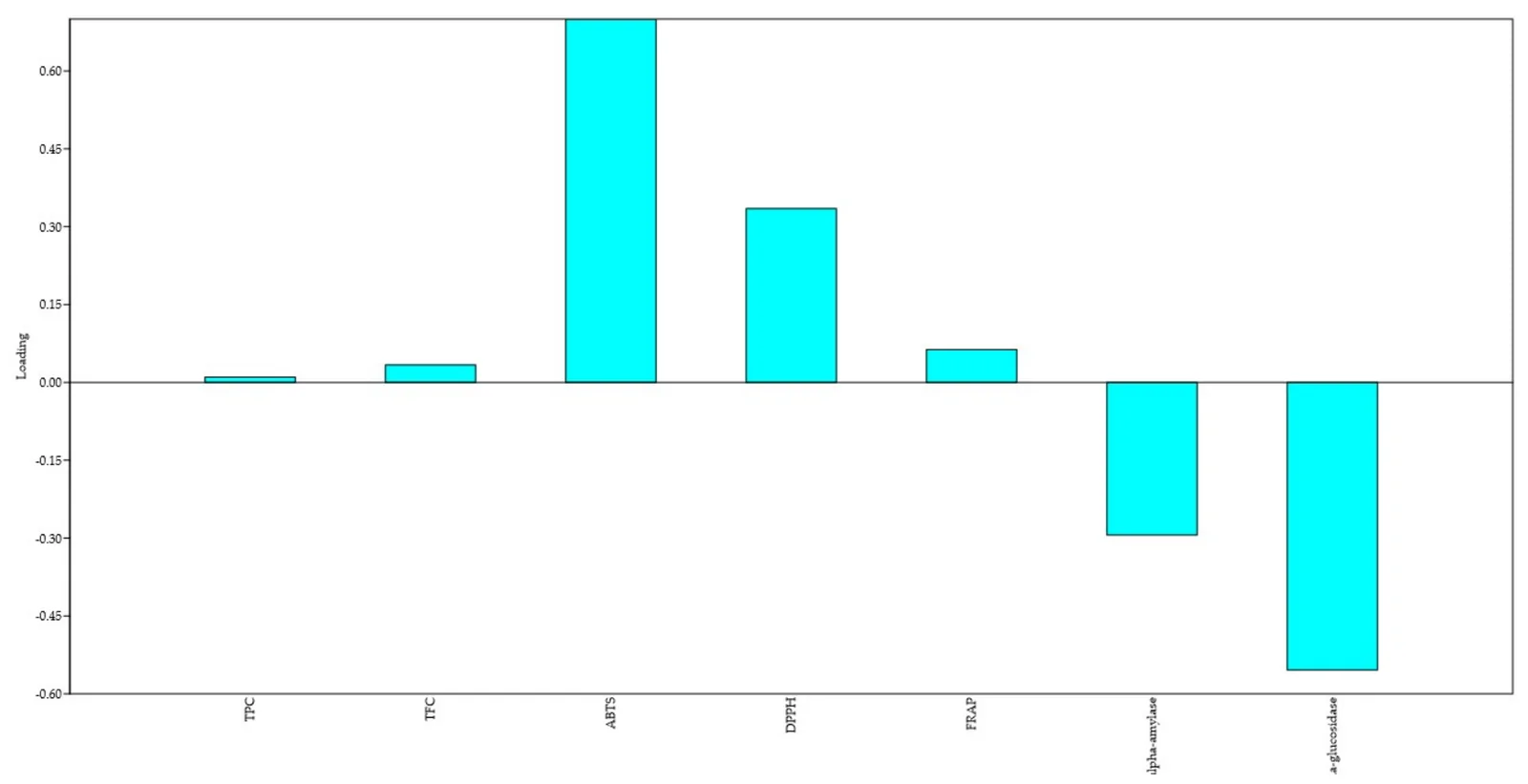
Principal component analysis revealing the influence of pectin and SCP supplementation on the bioactive and functional properties of watermelon jelly formulations, based on measurements performed using their 70% EtOH extracts. Abbreviations: W1—control jelly without SCP and pectin; W2—jelly supplemented with 0.5 g pectin; W3—jelly supplemented with 0.58 g pectin; W4—W1 supplemented with 0.215 g SCP; W5—W2 supplemented with 0.300 g SCP; W6—W3 supplemented with 0.215 g SCP; ABTS—2,2′-azino-bis(3-ethylbenzothiazoline-6-sulfonic acid); DPPH—2,2-diphenyl-1-picrylhydrazyl; FRAP—ferric reducing antioxidant power; TFC—total flavonoid content; TPC—total phenolic content.

Principal Component Analysis (PCA) (Figure 4) revealed a clear differentiation among the watermelon jelly formulations based on their bioactive and functional properties determined in their 70% EtOH extracts. The first two principal components explained 98.48% of the total variance, with PC1 accounting for 86.68% and PC2 for 11.80%, indicating that the first two components captured most of the variation among the analyzed samples.

The loading plot demonstrated that ABTS and DPPH assays had the strongest positive contribution to PC1, followed by FRAP, while TPC and TFC exhibited comparatively lower positive loadings. These findings suggest that antioxidant-related parameters were strongly associated with the positive side of PC1. In contrast, α-amylase and especially α-glucosidase inhibition showed negative loadings along PC1, indicating an inverse relationship between antioxidant activity and enzyme inhibitory potential. Among all variables, ABTS displayed the highest positive loading, whereas α-glucosidase exhibited the strongest negative loading, highlighting their dominant influence on sample separation.

The score plot demonstrated distinct clustering of the jelly formulations according to pectin and SCP incorporation. Formulations W1 and W2, prepared without SCP supplementation, were positioned in the upper right quadrant and were positively associated with α-glucosidase inhibitory activity. Sample W2, containing 0.5 g pectin, exhibited the highest positive PC2 value, suggesting that pectin addition influenced the measured functional properties compared to the control formulation W1.

Formulations enriched with SCP (W4–W6) were mainly distributed in the lower right quadrant, where they were more closely associated with α-amylase inhibition and antioxidant-related variables. Sample W4, corresponding to the SCP-enriched control formulation, exhibited the most negative PC2 score, indicating a stronger association with α-amylase inhibitory activity. Samples W5 and W6 occupied intermediate positions between antioxidant and enzyme inhibitoryvectors, suggesting that the combined incorporation of SCP and pectin contributed to a distinct profile of antioxidant and enzyme inhibitory properties. Notably, sample W5 showed the highest association with TFC, consistent with its elevated flavonoid content.

Interestingly, sample W3, formulated with the highest pectin concentration but without SCP supplementation, was strongly separated along positive PC1 values and positioned closer to ABTS and DPPH vectors, indicating a close relationship with antioxidant activity. This observation suggests that increased pectin concentration alone substantially influenced the antioxidant profile observed in the 70% EtOH extracts of the jelly formulations.

Overall, PCA confirmed that both pectin concentration and SCP supplementation were associated with differences in the measured functional properties of the jelly formulations. Pectin predominantly contributed to antioxidant-related properties, while SCP incorporation appeared to influence both antioxidant and enzyme inhibitory properties, particularly through enhanced flavonoid content and α-amylase inhibitory activity.

The PCA results support the hypothesis that pectin and SCP affected different functional attributes of of the developed formulations, as reflected in the properties measured in their 70% EtOH extracts. Pectin supplementation was primarily associated with antioxidant-related responses (ABTS, DPPH, and FRAP), whereas SCP supplementation showed a stronger association with α-amylase inhibitory activity and increased flavonoid content. The opposite orientation of the α-glucosidase vector relative to the antioxidant-related variables suggests that these biological responses were influenced by different formulation characteristics. These multivariate relationships are consistent with the individual univariate analyses and provide an integrated interpretation of the effects of pectin and SCP supplementation on the measured functional properties of the developed watermelon jelly formulations.

### 2.5. Limitations of the Study

A limitation of the present study is that the total protein and total carbohydrate contents of the formulated jellies were not determined. Therefore, any potential changes in nutritional composition resulting from SCP or pectin supplementation remain hypothetical and should be confirmed in future studies. Accordingly, the potential contribution of SCP to the nutritional value of the developed jellies is based on the known protein-rich composition of single-cell protein rather than on direct experimental evidence obtained in the present study. Future investigations should include comprehensive nutritional analyses to verify these compositional changes and to better characterize the nutritional profile of the developed formulations.

## 3. Materials and Methods

### 3.1. SCP Sample Preparation

#### 3.1.1. Media Preparation

By-products of potato (Wernsig Feinkost, Addrup-Essen/Oldenburg, Germany) and molasses (Nordzucker, Braunschweig, Germany) were used for SCP production. Potato peels were blended with distilled water at a 1:4 (*w*/*v*) ratio for 3 min using a blender (Blendtech Classic 575, Bad Homburg, Germany). The blend was then passed through a muslin cloth to obtain potato peel broth, which served as the fermentation medium. The potato peel broth was supplemented with molasses at a ratio of 70:30 (*v*/*v*) (70 mL potato peel broth and 30 mL molasses per 100 mL of fermentation medium) prior to sterilization. The medium was then autoclaved at 121 °C for 15 min before inoculation.

#### 3.1.2. Fermentation and SCP Production

In this study, the yeast used for fermentation was *Saccharomyces cerevisiae*, supplied as commercial dry baker’s yeast (Backfee, Vaihingen/Enz, Germany) and purchased from a local market in Germany. The inoculum suspension was prepared by aseptically adding 7 g of dried yeast to 5000 mL of the sterilized fermentation medium (1.4 g/L). Submerged fermentation was performed in 5000 mL conical flasks. The pH of the medium was adjusted to 6.0 with 1.0 N NaOH or 1.0 N H_2_SO_4_. The medium was sterilized at 121 °C for 15 min prior to inoculation. Fermentation was carried out at 25 °C under static conditions for 3 days. After fermentation, the yeast biomass was harvested by centrifugation at 4000 rpm for 15 min, washed twice with physiological saline, and freeze-dried for 48 h to obtain the SCP powder used for jelly formulation [43].

### 3.2. Sample Preparation

Fresh watermelon pulp and juice were obtained by homogenization and filtration of the edible part of the fruit. The homogenized sample was further concentrated to approximately 55% of its initial mass using a rotary evaporator RV 3 V (IKA-Werke, Staufen, Germany) under reduced pressure (−850 mbar) at 55 °C, a temperature selected to minimize thermal degradation of thermolabile compounds. The resulting concentrate was further processed into jelly formulations by the addition of sucrose (40 g per 60 g of sample), followed by short thermal treatment at 60 °C for 5 min, and pH adjustment to 3.0–3.5 using powdered citric acid.

Six watermelon jelly formulations (W1–W6) were prepared, including control samples without SCP and formulations differing in pectin (LM citrus pectin, Carbosynth, Compton, Berkshire, UK) and SCP incorporation. Formulation W1 represented the control jelly prepared without SCP and without pectin. Formulations W2 and W3 were prepared without SCP, but supplemented with 0.5 g and 0.58 g of pectin, respectively. To ensure meaningful comparison among formulations, all samples were normalized to the same final mass (8.8 g) by adjusting the amount of jelly base. SCP-enriched formulations were subsequently prepared by adding 0.215 g SCP into 8.585 g of W1 (W4), 0.300 g SCP into 8.500 g of W2 (W5), and 0.215 g SCP into 8.585 g of W3 (W6), resulting in final mass of 8.8 g for each formulation. Thus, formulations W4 and W6 contained identical amounts of SCP, whereas W5 contained a higher SCP level, enabling the evaluation of the combined effects of different pectin and SCP concentrations on the functional properties of the developed watermelon jellies. Each jelly formulation (W1–W6) was prepared once, yielding one independent sample for further analyses.

### 3.3. Chemicals

All enzymes and reagents were obtained from Sigma-Aldrich (Steinheim, Germany).

### 3.4. Extract Preparation

All jelly samples (W1–W6) were weighed (approximately 2 g) and extracted with 10 mL of 70% (*v*/*v*) ethanol (EtOH). The samples were sonicated in an ultrasonic bath (LEO Ultrasonic Co., Ltd., New Taipei City, Taiwan) for 15 min at 25 °C and subsequently centrifuged using an Eppendorf^®^ Centrifuge 5427R (Eppendorf SE, Hamburg, Germany). The resulting supernatants were collected and stored at 4 °C until further analyses. The extraction procedure corresponded to a final extract concentration of 200 mg/mL. One extract was prepared from each jelly formulation and used for all subsequent analyses.

### 3.5. Determination of Total Phenolic and Flavonoid Contents

#### 3.5.1. Total Phenolic Content (TPC)

The total phenolic content (TPC) of the 70% EtOH extracts prepared from the watermelon jelly formulations was determined using the Folin–Ciocalteu colorimetric assay according to Singleton et al. [44], adapted for microplate analysis. Gallic acid was used as the reference standard (0–100 µg/mL). Briefly, 25 µL of the sample extract or standard solution was mixed with 125 µL of 0.1 mol/L Folin–Ciocalteu reagent. After 10 min, 100 µL of 7.5% (*w*/*v*) sodium carbonate solution was added, and the reaction mixture was incubated at room temperature for 2 h. The absorbance was measured at 720 nm using a MultiScan Go spectrophotometer (Thermo Scientific, Waltham, MA, USA). The results were expressed as mg gallic acid equivalents per mL of extract (mg GAE/mL extract).

#### 3.5.2. Total Flavonoid Content (TFC)

The total flavonoid content (TFC) of the 70% EtOH extracts prepared from the watermelon jelly formulations was determined according to Chang et al. [45], adapted for microplate analysis, using quercetin as the reference standard. Briefly, 30 µL of each sample extract or standard solution was mixed with 90 µL of methanol, 6 µL of 0.75 mol/L aluminum chloride solution, 6 µL of 1 mol/L sodium acetate solution, and 170 µL of distilled water. The reaction mixture was incubated at room temperature for 30 min, after which the absorbance was measured at 415 nm using a MultiScan Go spectrophotometer (Thermo Scientific, Waltham, MA, USA).

The results were expressed as mg quercetin equivalents per mL of extract (mg QE/mL extract).

### 3.6. Antioxidant Activity Analysis

The antioxidant potential of the 70% EtOH extracts prepared from the formulated watermelon jellies was evaluated using complementary *in vitro* assays, including DPPH radical scavenging activity, ABTS radical cation decolorization assay, and ferric reducing antioxidant power (FRAP), following previously established procedures [46,47,48].

#### 3.6.1. DPPH Radical Scavenging Assay

DPPH radical scavenging (RSC) activity was determined according to the method of Espín et al. [46], using Trolox as the reference standard. A 90 μM DPPH working solution was freshly prepared on the day of analysis. The reaction mixture consisted of 10 μL of 70% EtOH sample extract or Trolox standard solution, 180 μL of methanol, and 60 μL of DPPH working solution. The mixtures were incubated in the dark at room temperature for 30 min, after which the absorbance was measured at 515 nm using a MultiScan Go spectrophotometer (Thermo Scientific, Waltham, MA, USA). The antioxidant capacity was expressed as mg Trolox equivalents per mL of extract (mg TE/mL extract).

#### 3.6.2. ABTS RSC Assay

The ABTS RSC activity was determined following the procedure of Arnao et al. [47]. The ABTS^•+^ stock solution was prepared by dissolving 7 mM ABTS and 2.45 mM potassium persulfate in distilled water and allowing the mixture to stand in the dark at room temperature for 16 h. A fresh working solution was prepared on the day of analysis by diluting 600 μL of the stock solution with 40 mL of 95% ethanol. The reaction mixture consisted of 290 µL of ABTS^•+^ working solution and 10 µL of 70% EtOH sample extract or Trolox standard solution. After incubation at room temperature for 5 min, the absorbance was measured at 734 nm using a MultiScan Go spectrophotometer (Thermo Scientific, Waltham, MA, USA). The antioxidant activity was expressed as mg Trolox equivalents per mL of extract (mg TE/mL extract).

#### 3.6.3. Ferric Reducing Antioxidant Power (FRAP) Assay

The ferric reducing antioxidant power (FRAP) assay was performed according to Benzie and Strain [48], using ascorbic acid as the reference standard. The FRAP reagent was freshly prepared by mixing an acetate buffer (pH 3.6), 10 mM TPTZ solution prepared in 40 mM HCl, and 20 mM FeCl_3_ × 6H_2_O solution in a ratio of 100:10:10 (*v*/*v*/*v*). The reaction mixture consisted of 10 µL of 70% EtOH sample extract or ascorbic acid standard solution, 225 µL of FRAP reagent, and 22.5 µL of distilled water. Following incubation at room temperature for 6 min, the absorbance was measured at 593 nm using a MultiScan Go spectrophotometer (Thermo Scientific, Waltham, MA, USA). The reducing antioxidant capacity was expressed as mg ascorbic acid equivalents per mL of extract (mg AAE/mL extract).

### 3.7. Enzyme Inhibitory Activity

The inhibitory effects of the 70% EtOH extracts prepared from the watermelon jelly samples on digestive enzymes associated with carbohydrate metabolism were evaluated through α-amylase and α-glucosidase inhibition assays.

#### 3.7.1. α-Amylase Inhibitory Activity

The α-amylase inhibitory assay was performed according to Yang et al. [49]. Briefly, 10 μL of 70% EtOH sample extract or acarbose solution was mixed with 90 μL of α-amylase solution (0.1 mg/mL, porcine pancreas, Type VI-B, EC 3.2.1.1, Sigma-Aldrich, St. Louis, MO, USA). After incubation at 37 °C for 10 min, 80 μL of 0.05% (*w*/*v*) soluble starch solution (Sigma-Aldrich, St. Louis, MO, USA) prepared in phosphate buffer (pH 6.9) was added, followed by a second incubation under the same conditions. The reaction was terminated by the addition of 100 μL of chilled HCl and 20 μL of Lugol’s iodine solution, and absorbance was measured at 620 nm. For the calculation of percentage inhibition, the control (A_control_) consisted of the reaction mixture containing 10 µL of 70% EtOH (the solvent used for sample extraction) instead of the sample extract. Sample and control corrections were included, and the corrected absorbance values (A_sample_ and A_control_) were calculated by subtracting the corresponding correction absorbance from the sample and control absorbance, respectively. Acarbose was used as the positive control and treated in the same manner, including the corresponding correction without enzyme. All reactions were performed in three technical replicates, and the mean corrected absorbance values were used to calculate the percentage inhibition according to the following equation:

% inhibition = (A_control_ − A_sample_)A_control_ × 100

#### 3.7.2. α-Glucosidase Inhibitory Activity

The α-glucosidase inhibitory activity was evaluated according to Palanisamy et al. [50]. Briefly, 10 μL of 70% EtOH sample extract or acarbose solution was mixed with 100 μL of phosphate buffer (pH 6.8) and 20 μL of α-glucosidase from *Saccharomyces cerevisiae* (0.4 U/mL working solution, EC 3.2.1.20, Sigma-Aldrich, St. Louis, MO, USA). After incubation at 37 °C for 15 min under continuous shaking (IKA KS 4000i control, IKA-Werke, Staufen, Germany), 20 μL of 5 mM *p*-nitrophenyl α-D-glucopyranoside (PNPG) was added as the substrate, followed by a second incubation under the same conditions. The reaction was terminated by the addition of 80 μL of 0.2 M Na_2_CO_3_, and absorbance was measured at 400 nm. For the calculation of the percentage inhibition, the control (A_control_) consisted of the reaction mixture containing 70% EtOH instead of the sample extract. Sample and control corrections were included, and the corrected absorbance values (A_sample_ and A_control_) were calculated by subtracting the corresponding correction absorbance from the sample and control absorbance, respectively. Acarbose was used as the positive control and treated in the same manner, including the corresponding correction without enzyme. All reactions were performed in three technical replicates, and the mean corrected absorbance values were used to calculate the percentage inhibition of α-glucosidase according to the following equation:

% inhibition = (A_control_ − A_sample_)A_control_ × 100

### 3.8. Statistical Analysis

Statistical analysis was performed using one-way analysis of variance (ANOVA), followed by Tukey’s post hoc test to determine significant differences among the analyzed samples. Each analytical assay was performed in triplicate (technical replicates) using a single extract prepared from each independently formulated jelly sample (*n* = 1 biological replicate per formulation). Results are expressed as mean ± standard deviation (SD). Differences were considered statistically significant at *p* < 0.05. All histograms were generated using OriginPro 8 (OriginLab Corporation, Northampton, MA, USA), while principal component analysis (PCA) was performed using PAST version 4.03 (Øyvind Hammer, Natural History Museum, University of Oslo, Oslo, Norway).

## 4. Conclusions

The present study demonstrated the successful development of low-sugar watermelon jelly formulations enriched with pectin and single-cell protein (SCP) using mild vacuum-assisted processing conditions designed to preserve bioactive compounds. The results demonstrated that formulation composition influenced the bioactive and functional properties of the developed jellies.

SCP supplementation and pectin incorporation influenced the phenolic and flavonoid composition of the developed watermelon jelly formulations. Although TPC values remained relatively stable among the investigated samples, a slight increase was observed following SCP addition, suggesting the contribution of bioactive metabolites and antioxidative constituents derived from microbial biomass. In contrast, TFC values varied significantly among formulations, with SCP-enriched samples, particularly sample W5, exhibiting the highest flavonoid content. All investigated 70% EtOH extracts prepared from the jelly formulations demonstrated considerable antioxidant potential, as confirmed by ABTS, DPPH, and FRAP assays, indicating that the applied processing conditions were compatible with the retention of measurable antioxidant activity. These findings suggest that the combined use of SCP and pectin may represent a promising strategy for the development of functional jelly products with potential bioactive and functional properties.

The 70% EtOH extracts prepared from the formulated jellies also exhibited *in vitro* inhibitory activity against α-amylase and α-glucosidase, indicating their potential to modulate carbohydrate digestion. Formulations containing both pectin and SCP generally showed stronger α-amylase inhibitory activity, whereas pectin-rich formulations exhibited greater α-glucosidase inhibition, suggesting that these ingredients contribute differently to enzyme inhibition.

Overall, the developed watermelon jellies represent promising sustainable functional food products with considerable antioxidant capacity and carbohydrate-hydrolyzing enzyme inhibitory activity. Given their demonstrated antioxidant activity, these formulations may serve as promising functional foods for future investigations into disorders associated with oxidative stress. Although oxidative stress has been implicated in the pathophysiology of chronic rhinosinusitis, the present study did not investigate this disease, and therefore no conclusions regarding potential benefits in chronic rhinosinusitis can be drawn. Future mechanistic, *in vivo*, and clinical studies are required to investigate this possibility. Furthermore, the incorporation of potato by-product-derived SCP as a sustainable ingredient supports the development of innovative functional foods aligned with current trends in sustainable nutrition and green processing technologies.

## Data Availability

All data are available within the article.
